# Enhancing research quality and reporting: why the *Journal of Comorbidity* is now publishing study protocols

**DOI:** 10.15256/joc.2014.4.46

**Published:** 2014-11-18

**Authors:** Susan M. Smith, Stewart W. Mercer, Jane Gunn, Marjan van den Akker, Martin Fortin

**Affiliations:** ^1^HRB Centre for Primary Care Research, Department of General Practice, RCSI Medical School, Dublin, Ireland; ^2^General Practice and Primary Care, Institute of Health and Wellbeing, University of Glasgow, Glasgow, UK; ^3^Primary Care Research Unit, Department of General Practice, University of Melbourne, Melbourne, Victoria, Australia; ^4^Department of Family Medicine, Research School Caphri, Maastricht University, Maastricht, The Netherlands; ^5^Department of Family Medicine, KU Leuven, Leuven, Belgium; ^6^Department of Family Medicine and Emergency Medicine, Université de Sherbrooke, Sherbrooke, Quebec, Canada, and Centre de santé et de services sociaux de Chicoutimi, Chicoutimi, Quebec, Canada

The *Journal of Comorbidity* was launched in 2011 and has since become established as a high-quality journal that publishes open-access, peer-reviewed articles, with a focus on advancing the clinical management of patients with comorbidity/multimorbidity. To further enhance research quality and reporting of studies in this field, the journal is now offering authors the opportunity to publish a summary of their study protocols – a move designed to generate interest and raise awareness in ongoing clinical research and to enable researchers to detail their methodologies in order that replication by scientific peers is possible.

Study protocols are a fundamental component of any study and provide insight into the thinking behind the study and how it will be conducted prior to its initiation. Protocols should meet the information needs of all stakeholders, including researchers, funding agencies, ethical reviewers, regulators, medical journals, and systematic reviewers [1]. They should provide a comprehensive overview of all aspects of the study conduct and contain a summary of roles and responsibilities, the rationale and study objectives, a full description of the study design, including study settings, sample size, eligibility criteria, interventions and timelines, data collection and statistical methodologies, data monitoring, and dissemination policies [1]. For studies investigating issues relating to comorbidity/multimorbidity, protocols should clearly define the comorbid or multimorbid conditions under investigation and outline any restrictions based on predetermined lists of conditions [2]; they should explain how any proposed interventions will address these conditions [3], and they should outline how the study will overcome issues relating to generalizability and/or external validity that can complicate comorbidity/multimorbidity studies [4]. When developing a protocol for a cohort study, ideally using a prospective design, consideration should be given to how the study might enhance understanding of the natural history and trajectory of the comorbid/multimorbid conditions, illness and treatment burden, patient quality of life and well-being, as well as the quality, safety, and cost-effectiveness of care [5].

## Why publish study protocols?

Publishing study protocols serves several important purposes. Publication increases research quality and transparency, encourages communication and collaboration between research teams, helps to disseminate contemporary ideas about study design and data analysis, avoids research duplication, and may assist study recruitment.

The availability of a published final protocol allows subsequent authors and reviewers to cross-reference the study details, assists in presenting the final results, aids systematic evaluation of the data, and discourages reporting bias. Importantly, it enables readers to compare the original aims and design of the study with the final reported outcomes, thereby reducing the potential for unexplained *post-hoc* revisions of the study goals and methodologies – a problem that appears to affect the reporting of both observational studies and randomized controlled trials.

Publication of study protocols in peer-reviewed journals rightfully engenders the expectation that the results will eventually be disseminated, and encourages investigators to report their findings in a timely and appropriate way. Last, but not least, we believe that publication of study protocols recognizes the substantial amount of work that is required for their development, affording research professionals, who may otherwise remain unseen, an opportunity to promote their work.

## Publishing your study protocol in the Journal of Comorbidity

- The *Journal of Comorbidity* will peer review protocols unless the study has received approval from an institutional review board (IRB)/ethics committee and has received peer-reviewed grant support from a major extramural funding body
- The *Journal of Comorbidity* endorses the SPIRIT (Standard Protocol Items: Recommendations for Interventional Trials) Statement [1]. Authors are required to follow these guidelines when preparing their protocol, with specific attention being paid to the challenges of comorbidity/multimorbidity research.

## Types of protocol

- Protocols that describe the design of planned or ongoing research will be considered for publication
- Protocols for any study design will be considered, including observational, qualitative, exploratory studies, experimental studies, and systematic reviews. We encourage the submission of protocols for studies that will evaluate specific aspects of clinical trial design
- Study protocols should generally provide a detailed account of the hypothesis, rationale, contextual background, design/methodology, statistical considerations, and analysis of the proposed research
- Study protocols should be presented with sufficient detail to enable replication of the methods by others
- Full details of the requirements for submitting a protocol are described in the journal’s guidelines to authors, which can be found online (www.jcomorbidity.com).

## Benefits

- Open access – freely available online for readers worldwide
- Rapid publication
- High visibility
- Authors retain copyright
- Adequate space for comprehensive protocol description^1^
- Authors are not required to submit subsequent reports of their study to the journal; however, the editors encourage the authors to consider doing so.

## Submit your protocol to the Journal of Comorbidity

- We invite all researchers involved in work that is pertinent to comorbidity/multimorbidity to submit their study protocols to the *Journal of Comorbidity*
- To submit your protocol to the *Journal of Comorbidity*, go to the submissions page to register or login (http://www.jcomorbidity.com/index.php/test/user/register).

## About the Journal of Comorbidity

The *Journal of Comorbidity* is an international, open-access, peer-reviewed journal for the pathophysiology, diagnosis, prevention and management of patients with comorbidity/multimorbidity. The journal currently publishes original clinical and experimental research articles, treatment guidelines/policies, editorials, commentaries, and critical review articles, as well as proceedings of congresses. Preference is given to articles that advance the clinical management of patients. The Editorial Board also welcomes ideas and suggestions for *special issues* dedicated to a unique theme. The journal provides immediate, permanent open access to its content on the principle that making research freely available to the public supports a greater global exchange of knowledge.

## Footnotes

1 Authors should refer to the journal guidelines when preparing their protocol for submission
